# Personality Changes and Staring Spells in a 12-Year-Old Child: A Case Report Incorporating ChatGPT, a Natural Language Processing Tool Driven by Artificial Intelligence (AI)

**DOI:** 10.7759/cureus.36408

**Published:** 2023-03-20

**Authors:** Vidya Puthenpura, Siddhi Nadkarni, Michael DiLuna, Kimberly Hieftje, Asher Marks

**Affiliations:** 1 Pediatric Hematology and Oncology, Yale School of Medicine, New Haven, USA; 2 Pediatrics, Yale School of Medicine, New Haven, USA; 3 Neurosurgery, Yale School of Medicine, New Haven, USA

**Keywords:** artificial intelligence, chatgpt, low grade glioma, pediatric neuro-oncology, pediatric oncology

## Abstract

Low grade gliomas (LGGs) are the most common type of brain tumors diagnosed in children. The presentation of intracranial tumors in pediatric patients is varied and diverse. The early identification and treatment of LGGs are important to achieve favorable outcomes. Although personality changes can be a symptom of intracranial tumors, they are rarely the only main presenting feature. In addition to central nervous system (CNS) tumors, personality changes can be associated with psychological and endocrine conditions, contributing to a broad differential diagnosis. Because symptoms such as personality changes have the potential to be missed, communication between family members and clinicians is imperative to identify these symptoms early. We report the case of a 12-year-old child who presented with personality changes as her main symptom and was found to have an intracranial neoplasm. This case report integrates original author writing with output from ChatGPT, a natural language processing tool driven by artificial intelligence (AI). In addition to the case itself, this report will explore the benefits and drawbacks of using natural language AI in this context.

## Introduction

Central nervous system (CNS) tumors are the most common solid tumors in the pediatric population [1]. The incidence of pediatric CNS tumors is approximately 5.4 diagnoses per 100,000 [2]. Among those diagnosed with a CNS tumor between the ages of 0 and 14, the average annual age-adjusted mortality rate is 0.7 per 100,000, such that CNS tumors are the primary cause of pediatric cancer death [3]. Low-grade gliomas (LGGs) are a type of brain tumor that affect primarily children and adolescents [4]. They are considered low-grade tumors as they grow slowly and have a relatively low potential to spread to other parts of the brain or the spinal cord [4]. LGGs are the most common type of brain tumor in children, accounting for approximately 40%-50% of all pediatric brain tumors [4]. Although histology has long been used to classify LGGs, molecular diagnostics, specifically the identification of the up-regulation of the RAS-mitogen-activated protein kinase pathway, have led to targeted molecular therapies for LGGs [1].

The LGGs can cause a range of symptoms depending on the location of the tumor within the brain. Some common symptoms associated with LGGs in children include headaches, seizures, and vomiting. Other neurological symptoms may include changes in behavior, personality, or cognition, as well as partial weakness or numbness. These symptoms can be caused by the tumor itself or by the pressure exerted on surrounding structures in the brain. In some cases, LGGs can also cause visual disturbances or hormonal imbalances. It is important for parents and caregivers to be aware of these symptoms and seek medical attention if their child experiences any of them, especially if they are new or worsening over time. We report the case of a 12-year-old girl with an intracranial tumor who presented with personality changes as her primary initial symptom, a presentation not well described in the literature that was successfully managed with gross total resection of the mass.

The abstract, introduction, discussion, and conclusion have been written with the integration of original author writing and ChatGPT output. A separate discussion will describe the authors’ experience of using ChatGPT in this context.

## Case presentation

**Figure 1:**
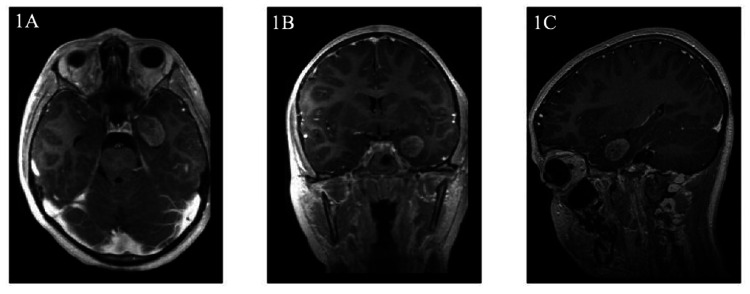
T1-weighted MRI of patient's intracranial tumor. T1-weighted MRI characterizing the mass as a 1.7 cm x 1.7 cm x 2.6 cm left medial temporal lobe enhancing lesion in the (A) transverse, (B) coronal, and (C) sagittal views.

A 12-year-old previously healthy girl presented to pediatric neurology with two months of staring spells and one year of personality changes consisting of aggressive outbursts, periods of anxious and angry behavior, and trouble with focusing at school. Neuropsychological testing was performed but yielded limited results. Approximately one month prior to presentation, psychiatry was consulted and the patient was started on sertraline for mood dysregulation with minimal improvement. The staring spells occurred two to three times per day with increasing frequency, with each episode lasting up to 30 s. During these episodes, she would remain conscious but minimally responsive and would report having no memory of the episodes after they occur. Her neurological exam was completely intact. MRI of the brain performed by neurology in the outpatient setting showed a left medial temporal intracranial mass measuring 1.7 cm x 1.7 cm x 2.6 cm left medial temporal lobe enhancing lesion, adjacent to the amygdala and entorhinal cortex, exerting mass effect on the left temporal horn and displacing the hippocampus and temporal sulcus laterally (Figure 1).

Prior to gross total resection of the tumor, a continuous EEG was performed to identify potential epileptiform findings or seizures. No seizures were noted, but occasional left anterior-mid temporal sharp waves were observed, suggestive of epileptic potential within this underlying region. Accordingly, the patient was started on brivaracetam while continuing on sertraline. After gross total resection of the tumor, pathology revealed an astroglial neoplasm with Ki67 < 5%, MGMT unmethylated, and BRAF-KIAA1549 fusion and BRAF V600E negative [5-7]. Based on these characteristics, the tumor was characterized to be low-grade, not requiring further treatment, with a low likelihood of recurrence [4]. Following surgical resection, the patient’s behavioral changes were noted to be significantly less severe and frequent. She was weaned off the brivaracetam and serial scans every three months through the first year remained stable with no signs of recurrence.

## Discussion

Case discussion

Personality changes, the main presenting symptom in this case, are associated with a broad differential diagnosis. The differential diagnosis for this presentation can be grouped into three main categories: psychosocial, endocrine-related, and neurological. In addition, given that the patient is an adolescent, puberty can, itself, be associated with personality changes [8]. The intense nature of the patient’s personality changes, however, combined with staring spells, made symptoms of pubertal development a less likely cause. Hypothyroidism, characterized by low levels of thyroid hormone, can present with affective disorders and psychosis. Although hypothyroidism is more prevalent in women, it tends to affect older women, as opposed to children and adolescents [9]. Additionally, hyperthyroidism can present with personality changes in the form of anxiety [10].

A review of 200 pediatric brain tumor cases found that patients presented with educational and behavioral problems as the first presenting symptom in 10% of cases and occurring at any time in 44% of cases [11]. Another study, which sought to characterize the symptoms of brain tumors diagnosed in the ED, found that 17.2% of pediatric patients with brain tumors had behavioral or school change as reported symptoms [12]. A 2010 literature review assessed the association between brain tumors and psychiatric symptoms, and found that the symptoms often were dependent on the location of the tumor [13]. 

Diagnosis of LGGs with personality changes as the only symptom can be challenging. This is because these symptoms can be vague, non-specific, and may be confused with other psychiatric or behavioral disorders. The presence, however, of sudden and/or extreme personality changes, especially in the absence of other symptoms, should prompt a thorough evaluation to rule out a CNS lesion, including brain imaging. CT scans are often performed initially, but MRI is the preferred modality for evaluating the brain in these cases due to its higher sensitivity and specificity.

The management of LGGs is multidisciplinary and depends on several factors, including the size and location of the tumor, extent of initial resection, the child's age and overall health, and the molecular drivers of the tumor. Gross total resection is the primary treatment for LGGs, which is associated with 10-year overall survival rates of 90% or greater, however, various cytotoxic chemotherapies, radiation, and targeted therapies have their roles [4]. Because this tumor was completely resected, no further treatment was deemed necessary. Further monitoring via serial scans every three months for the first year is the standard of care, with decreasing frequency moving forward.

For progressive LGGs that are not amenable to gross total resection, chemotherapy, consisting of a combination of carboplatin and vincristine is frequently the frontline approach, with a three-year progression-free survival of 68%, although the rise of carboplatin hypersensitivity reactions must be monitored [14-15]. Second- and third-line therapies include vinblastine, bevacizumab, temozolomide, and radiation [16-18]. More recently, targeted therapies, including MEK inhibitors and BRAF inhibitors, can be considered for LGGs that are BRAF-KIAA1549 fusion (MEK inhibitors only) or BRAF V600E positive [19].

Discussion of utilizing ChatGPT

**Figure 2:**
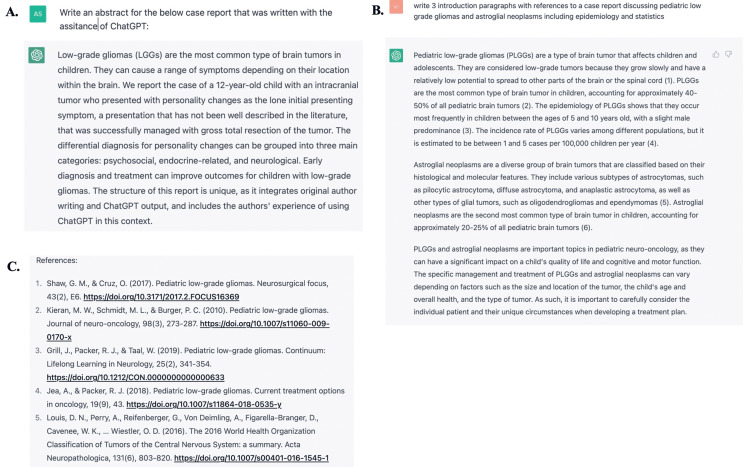
Excerpts from ChatGPT: prompts and output. Excerpts from ChatGPT with (A) abstract provided by ChatGPT after input of the manuscript (B) example prompt and output, and (C) references cited by ChatGPT that were utilized to respond to the prompt.

This case report was written with the assistance of ChatGPT, a natural language processing tool driven by artificial intelligence (AI). All relevant information about the patient, including the case presentation, diagnostic test results, and treatment information were collected by the authors. This informed the development of carefully crafted prompts that were inputted through ChatGPT. The outputted texts were reviewed for accuracy and relevance to the case report. The prompts and the output from ChatGPT are included in Appendix 1. The output from ChatGPT was then integrated into the original manuscript written by the authors. The original introduction and discussion prior to the integration of information from ChatGPT are provided in Appendices 2 and 3, respectively. Upon completion of the manuscript, the authors asked ChatGPT to generate an abstract after pasting the body into ChatGPT’s query line. This offered insight into structuring the final abstract (Figure 2A). Through the above processes, we discovered that ChatGPT can be used as a powerful tool to assist in writing case reports and streamline the process. AI programs such as ChatGPT can help with general structure and broad themes of case reports, however, we did also discover some serious concerns with the use of ChatGPT in medical manuscript writing.

We found multiple instances where information was incomplete or difficult to interpret without already having an expert knowledge of the subject matter. In this situation, without having background knowledge of pediatric neuro-oncology, and specifically about LGGs, it would have been difficult to evaluate what information provided by ChatGPT was relevant to the case report or important to highlight. In addition, the authors discovered that the references included by ChatGPT (Version February 13, 2023) did not always exist or were not accurate. For example, Figure 2B shows one prompt that was used within ChatGPT to incorporate into the introduction of this case report. The figure shows in-text citations that correspond to the references listed in Figure 2C. Utilizing PubMed(R), a database through the National Library of Medicine, the authors verified that the first four references, including the digital object identifier (DOI) numbers, do not exist. This is extremely concerning. When ChatGPT was queried about this discrepancy, ChatGPT explained:

“I apologize again for the mistake in my previous responses. As an AI language model, I do not provide fake references intentionally. I am programmed to retrieve information from reliable sources to the best of my ability, but sometimes mistakes can happen due to errors in my training data or retrieval process. I appreciate your feedback and will do my best to ensure the accuracy of my responses in the future.”

In addition, concerns about ChatGPT’s usage for plagiarism calls for an immediate need to ensure that AI is harnessed in a way that is appropriate and augments original text. Any utilized AI technology should be cited and acknowledged in a manuscript. To take this a step further, we would propose that facts, findings, and conclusions drawn and developed utilizing AI be specifically called out in the paper through varied typeface or annotation. In addition, we also propose the development of an algorithm to measure the degree to which a scholarly work is “AI informed” and make it a requirement to include this measure in any paper utilizing natural language processing tools or other AI driven technologies.

## Conclusions

This report presented a case of a child with an intracranial LGG with personality changes as her only presenting symptom. The differential diagnosis for personality changes can be quite broad, and it is important for healthcare providers to consider an oncologic etiology. The authors were assisted by ChatGPT in outlining this case report. They found that while ChatGPT had its strengths, such as providing a structure and identifying broad themes to format the report, there are significant challenges that need to be considered when using ChatGPT or other AI-based technology for medical manuscript writing.

## Figures and Tables

**Figure 3:**
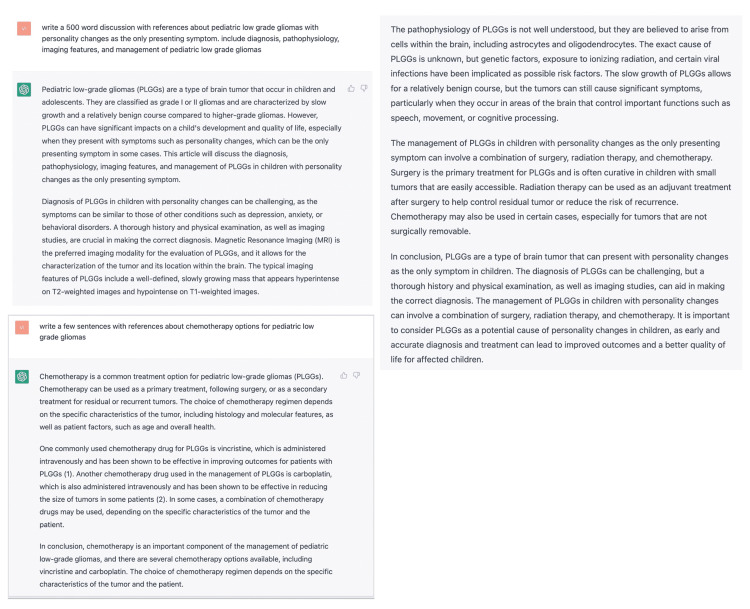
Screen capture of prompts and outputs used from ChatGPT.

## Appendices

Appendix 1: Screen capture of prompts and outputs used from ChatGPT 

Appendix 2: Original introduction written by the authors

Central nervous system (CNS) tumors are the most common solid tumors in the pediatric population [1]. The incidence of pediatric CNS tumors is approximately 5.4 diagnoses per 100,000 [2]. Among those diagnosed with a CNS tumor between the ages of 0 and 14, the average annual age-adjusted mortality rate is 0.7 per 100,000, such that CNS tumors are the primary cause of pediatric cancer death [3]. Low-grade gliomas (LGGs) comprise approximately 30%-50% of all pediatric brain tumors [4]. There are many subclassifications of LGGs based on histology and include astrocytic, oligodendroglial, and mixed glial-neuronal tumors. Although histology has long been used to classify LGGs, molecular diagnostics, specifically the identification of the up-regulation of the RAS-mitogen-activated protein kinase pathway, have led to targeted molecular therapies for LGGs [1].

Most pediatric low-grade tumors are slow-growing and not malignant. Although LGGs can be found anywhere in the CNS, they are most commonly found in the posterior fossa. Hemispheric, cerebral gliomas, and gliomas of the deep midline structures each account for 10%-15% of all CNS tumors, optic pathway gliomas account for 5% of all CNS tumors, and brain stem gliomas account for 2%-4% of all CNS tumors [4]. The clinical presentation for low-grade tumors consists of generalizing and localizing symptoms. Generalizing symptoms, which are often a result of increased intracranial pressure due to ventricle obstruction, include headaches, difficulty walking, nausea, vomiting, and cognitive challenges. Focal or localizing symptoms include seizures, aphasia, and visual field defects [20]. About half of all children with low-grade tumors will have had symptoms for 6 months or longer before diagnosis [21]. Low-grade tumors can often be diagnosed via MRI imaging and are often hyperintense on T2-weighted and hypointense on T1-weighted images. They may or may not enhance with contrast [4].

We report the case of a 12-year-old child with an intracranial tumor who presented with personality changes as the main presenting symptom, a presentation that has not been well documented in the literature, successfully managed with gross total resection of the tumor.

Appendix 3: Original discussion written by the authors

Personality changes, the main presenting symptom in this case, are associated with a broad differential diagnosis. The differential diagnosis for this presentation can be grouped into three main categories: psychosocial, endocrine-related, and neurological.

Psychiatric disorders such as bipolar disorder, anxiety, and schizophrenia, have been associated with personality changes. Research has shown that children with pediatric bipolar disorder have more severe personality changes, compared to adults, and are predisposed to neuroticism, which can enhance feelings of anger, anxiety, and emotional instability [22]. Although a psychiatric diagnosis was considered for this patient, the staring spells raised greater suspicion for an intracranial process.

Furthermore, noting the patient’s adolescent age, puberty can also be associated with personality changes [8]. Although more research is needed, hormonal changes are believed to be responsible for many of the personality changes that are attributed to puberty, such as mood swings and emotional lability. In addition to influencing early brain development, hormones may influence personality and behavior by acting on regions of the central nervous system that are critical to the identification and processing of sensory information [23]. The intense nature of the patient’s personality changes, however, combined with staring spells, makes symptoms of pubertal development a less likely cause.

Thyroid dysfunction can also be associated with personality changes. Hypothyroidism, characterized by low levels of thyroid hormone, can present with affective disorders and psychosis. Although hypothyroidism is more prevalent in women, it tends to affect older women, as opposed to children and adolescents [9]. Additionally, hyperthyroidism can present with personality changes in the form of anxiety [10].

Although psychosocial and endocrine diagnoses can present with personality changes, a neurological diagnosis better incorporates the presentation of staring spells. Staring spells can be indicative of absence seizures, which are typically seen in children ages 4-12 years. Due to their brief nature and tendency to be mischaracterized as lack of attention or misbehavior, absence seizures can occur for months before diagnosis [24].

A review of 200 pediatric brain tumor cases found that patients presented with educational and behavioral problems as the first presenting symptom in 10% of cases and occurring at any time in 44% of cases [11]. Another study, which sought to characterize the symptoms of brain tumors diagnosed in the ED, found that 17.2% of pediatric patients with brain tumors had behavioral or school change as reported symptoms [12]. A 2010 literature review assessed the association between brain tumors and psychiatric symptoms, often depending on the location of the tumor [13]. The authors note that frontal lobe tumors more commonly present with depression, while temporal lobe tumors often present with psychosis and schizophrenia-like symptoms. Although the broad range of personality and behavioral changes that can arise from brain tumors has not been fully characterized, a 2017 study found that the most common neuropsychological symptoms of intracranial tumors are anxiety, agitation, irritability, depression, and sleep disturbances [25].

The standard approach for this type of tumor, given its size and location, is surgical intervention with the goal of gross total resection, which is associated with 10-year overall survival rates of 90% or greater [4]. Because this tumor was characterized as a low-grade tumor, no further treatment is required following gross total resection and the likelihood of recurrence is low. Further monitoring via serial scans every three months for the first year is the standard of care.

Although gross total resection of the tumor is often the ideal treatment approach, total resection of the tumor is not always possible due to the location and size of the tumor. Other treatment options include subtotal resection and biopsy only or no surgical intervention [4]. For progressive low-grade gliomas that are not susceptible to resection, chemotherapy, consisting of a combination of carboplatin and vincristine, is the frontline approach, with a three-year progression-free survival of 68%, although the rise of carboplatin hypersensitivity reactions must be monitored [14-15].  Although the tumor in this case was BRAF-KIAA1549 fusion negative, targeted therapies should be considered for positive tumors [19].
